# Integrating Boronic Esters and Anthracene into Covalent Adaptable Networks toward Stimuli-Responsive Elastomers

**DOI:** 10.3390/polym14061104

**Published:** 2022-03-10

**Authors:** Zhiyong Liu, Youwei Ma, Yixin Xiang, Xianrong Shen, Zixing Shi, Jiangang Gao

**Affiliations:** 1 Department of Polymer Materials and Engineering, School of Chemical and Environmental Engineering, Anhui Polytechnic University, Wuhu 241000, China; liuzhiyong@ahpu.edu.cn (Z.L.); xiangyixin@ahpu.edu.cn (Y.X.); shenxianrong@ahpu.edu.cn (X.S.); 2 State Key Laboratory for Metal Matrix Composite Materials and Shanghai Key Laboratory of Electrical Insulation & Thermal Ageing, School of Chemistry and Chemical Engineering, Shanghai Jiao Tong University, Shanghai 200240, China; mayouwei1995@sjtu.edu.cn

**Keywords:** stimuli-responsive, dynamic covalent bonds, SBS, anthracene, boronic esters

## Abstract

Stimuli-responsive polymer materials have a promising potential application in many areas. However, integrating multi-stimuli into one elastomer is still a challenge. Here, we utilized boronic esters and anthracene to prepare a cross-linked poly(styrene-butadiene-styrene) (SBS) which was endowed with responsiveness to three stimuli (light, heat, and alcohols). SBS was first functionalized with a certain amount of dihydroxyl groups via a thiol-ene “click” reaction between unsaturated double bonds in PB block and thioglycerol. Then, 9-anthraceneboronic acid was applied to form a cross-linked SBS network upon heat and ultraviolet radiation (λ = 365 nm). The prepared elastomer was demonstrated to be stimuli-responsive based on the dynamic nature of boronic esters and the reversible dimerization of anthracene. In addition, the mechanical properties of the elastomer could be regulated continuously owing to the stimulus responsiveness to ultraviolet or heat.

## 1. Introduction

Stimuli-responsive polymer materials are in a class of “smart” materials which could respond to external stimuli, such as temperature, pH, chemicals, stress, light, electricity, magnetism, etc. [1,2,3,4]. Among these stimuli, temperature- and light-responsive mechanisms have been considerably researched because of their convenience and efficiency in many applications [5,6,7]. The light, especially, has excellent properties, including environmental protection, remote control, and instantaneity. The interest in these materials has increased yearly due to their promising potential applications in catalysis, tissue engineering, biosensors, and drug delivery [8,9,10,11,12]. Investigations of the response to single stimulus are highlighted. Dual- or multi-stimuli-responsive polymers, which are capable of adapting to changes in response to two or more stimuli, are synthetically challenging but are an emerging interest [13,14,15,16]. The coexistence of multi-triggers could greatly enhance the versatility of these materials in many applications. One route to prepare these materials is introducing multi-stimuli-responsive components into the polymer matrix. Among the reported components, anthracene could undergo photodimerization via a [4+4] cycloaddition reaction when radiated under ultraviolet (λ = 365 nm). Corresponding de-dimerization is achieved simply by a different irradiating wavelength (λ = 254 nm) or by heat (above 150 °C) [17,18,19]. It is believed that anthracene is ideal for preparing stimuli-responsive polymers attributed to its light and temperature responsiveness.

Generally, adequate mechanical properties are also indispensable for the practical application of stimuli-responsive polymer materials. Commercially available poly(styrene-butadiene-styrene) (SBS) constitutes an important class of thermoplastic elastomers and has been widely used, owing to its excellent elasticity and thermoplasticity. However, the excellent properties of SBS are limited, only being applied in tires, shootwears, compatibilizers, and membranes, etc. There is a need to explore the potential applications of SBS in smart polymeric areas. SBS was composed of two polystyrene (PS) hard blocks and one polybutadiene (PB) soft block. The blocks could self-assemble into highly ordered microdomain morphologies with characteristic dimensions on the molecular scale, while the PS’s morphology served as physical cross-links. Unfortunately, the solvent resistance, thermal stability, and elastic resilience of SBS were found to be poor, resulting from physical cross-links which are unfavorable for chemical- or temperature-responsive polymers. To improve its dimensional stability, various modified SBSs were reported. However, as is known, the chemical cross-links formed by vulcanization inevitably destroy the thermoplasticity. Therefore, a new type of cross-linked network needs to be designed for SBS to remove this awkward situation.

Over the past few years, polymer networks possessing reversible covalent cross-links, named as covalent adaptable networks (CANs), have been reviewed in detail [20,21,22,23,24,25]. The key point of this concept was the incorporation of dynamic covalent bonds (DCBs) to form covalent networks. Nowadays, various DCBs were found, including disulfide bond [26,27,28], imine [29,30,31], transamination [32,33,34], and boronic esters [35,36,37,38], etc. Among these DCBs, boronic esters were interesting because of the facile preparation and inherent stability. Boronic esters could be formed by the reaction of boronic acids with diols in anhydrous organic solutions. When the cross-links were boronic esters, the network could be adapted to a new one upon heat. Moreover, the network changed by alcohols which could be used as an external stimulus for responsive materials as well.

The primary objective of the present study was to construct a stimuli-responsive cross-linked SBS network while the intrinsic properties, such as the elasticity and processibility of SBS, were retained. To obtain the desired cross-linked SBS, boronic esters were incorporated to construct CANs, and anthracene was also referred for its light and heat responsiveness. It was anticipant that the designed stimuli-responsive elastomer would render interesting properties that could have potential applications in smart polymeric materials.

## 2. Experimental Section

### 2.1. Materials

Poly(styrene-butadiene-styrene) (SBS) (styrene 30 wt%, average M_w_ = 140,000) was purchased from Sigma-Aldrich. Thioglycerol, 9-anthraceneboronic acid, and 2-methyl-4-(methylthio)-2-morpholinopropiophenone (I907) were supplied by Sinopharm Chemical Reagent Co., Ltd. (Beijing, China). All other reagents were commercial chemicals and used as received, without further purification.

### 2.2. The Synthesis of Dihydroxyl Groups Functionalized SBS

Dihydroxyl groups of functionalized SBS were synthesized by the thiol-ene “click” reaction. The brief procedure was as follows: SBS (5 g), thioglycerol (0.28 g), and photoinitiator I907 (0.01 g) were mixed in chloroform (300 mL) to form a homogeneous solution. Then, the solution was stirred and radiated under a UV lamp (365 nm, 100 mw) for 3 h at room temperature. After the reaction was completed, large amounts of solvent were firstly removed by a rotary evaporator, and the residual was poured into ethanol. The precipitate was washed with ethanol three times and dried under vacuum for a constant weight at 40 °C. For convenience, SBS functionalized with the dihydroxyl groups was denoted as SBS–OH.

### 2.3. Preparation of Cross-Linked SBS Networks (SBS–Ban_x_)

A specified amount of 9-anthraceneboronic acid (0.17 g) dissolved in DMF was added into a toluene solution of SBS–OH (2 g). After being stirred for 20 min, the mixture was cast into a PTEF mold (100 mm × 100 mm × 10 mm) and evaporated in an oven at 80 °C for 3 d. While the film was radiated under UV (365 nm) for a certain time, cross-links were formed as a result of the photodimerization of anthracene. Then, the resulted network was denoted as SBS–BAn_x_, in which “x” is the ultraviolet radiation time.

### 2.4. Reprocessing of SBS–BAn_x_

Reprocessing of SBS–BAn_x_ under pressure was conducted on a press vulcanizer. The SBS–BAn_x_ was, firstly, cut into pieces, then piled up into a steel mold (80 mm × 80 mm × 1 mm) and reprocessed under 10 MPa at 120 °C for 60 min, or at 160 °C for 30 min.

### 2.5. The Calculation of Gel Fraction and Swelling Ratio

Pieces of SBS–BAn_x_ film (m_0_) were submersed in xylene in a sealed bottle and stirred slowly for 3 d at 80 °C. Then, the swelling samples were taken out and wiped by filter paper, followed by weighing (m_1_). Subsequently, the samples were dried in a vacuum oven at 80 °C to a constant weight (m_2_). The swelling ratio (SR) and gel fraction (GF) could be calculated according to equations, as follows:

(1) $$\mathrm{SR}\;=\frac{m_{1}-m_{2}}{m_{2}}$$

(2) $$\mathrm{GF}\;=\frac{m_{2}}{m_{0}}$$

where m_0_ is the weight of the film before immersion in the solvent, and m_1_ is the weight when it was taken out. m_2_ is the weight of the sample after drying.

### 2.6. Characterizations

Nuclear magnetic resonance spectroscopy (^1^H NMR) was recorded on samples dissolved in CDCl_3_ with a Bruker AV500 MHz spectrometer (Bruker BioSpin Corp. Billerica, MA, USA) at room temperature. Chemical shifts were referenced to tetramethylsilane (TMS). The dihydroxyl modification ratio in SBS–OH was determined on the basis of ^1^H NMR integrals.

SEM images were recorded on a JEOL JSM-6700F field emission scanning electron microscope (Tokyo, Japan), operating at 5 kV.

Element analysis (EA) was performed on a Vario EL Cube (Langenselbold, Germany).

The mechanical properties of prepared films were determined with an Instron 4465 electronic universal testing machine (Instron Corporation, MA, USA) at a crosshead speed of 10 mm min^−1^. Dumbbell-shaped specimens were cut according to GB/T528 (overall length: 50 mm; inner width: 4 mm). At least five specimens per experimental point were tested by all mechanical measurements to obtain reliable values.

Dynamic mechanical analysis (DMA) was performed on a DMA Q800 (TA Instruments, New Castle, DE USA). The temperature dependence of the storage modulus (G’) and the loss modulus (G’’) was recorded from −100 °C to 200 °C. The frequency used was 1.0 Hz at a heating rate of 5 °C min^−1^.

UV–vis spectra of the samples were recorded on a UV-2550 spectrophotometer. (Shimadzu, Japan).

## 3. Results and Discussion

**Scheme 1:**
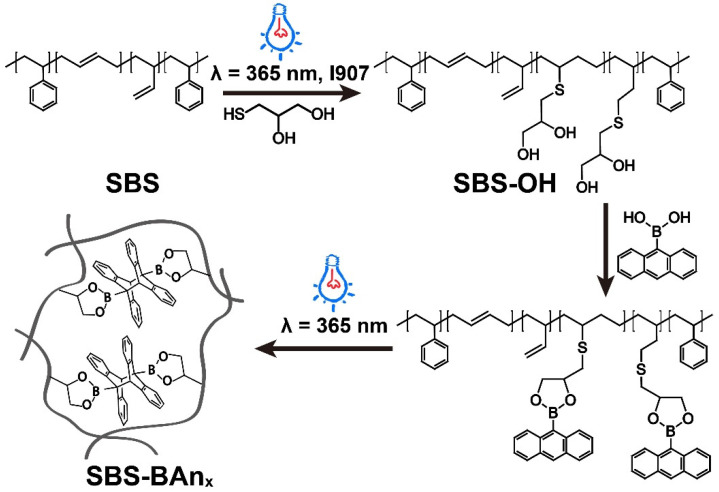
The synthesis route of the cross-linked elastomer.

Aiming to fabricate a stimuli-responsive polymer material with excellent mechanical properties, commercial SBS was selected as the polymeric matrix. To improve the thermal stability and solvent resistance of SBS without sacrificing recyclability, DCBs were involved to form CANs. Considering unsaturated double bonds were presented in the PB block, though thiol-ene “click” chemistry was a preferred tool because of its high efficiency and selectivity. Therefore, SBS functionalized with dihydroxyl groups, denoted as SBS–OH, was prepared by reaction with thioglycerol. Then, 9-anthraceneboronic acid was utilized to introduce the two dynamic components, including boronic esters and anthracene. The synthesis route of the cross-linked elastomer is shown in Scheme 1.

**Figure 1:**
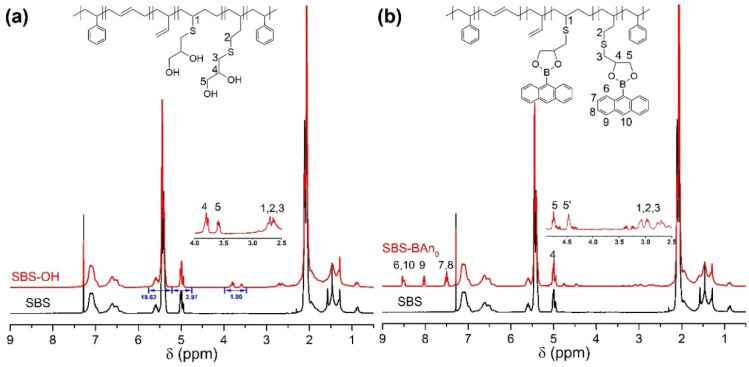
^1^H NMR spectra of (**a**) SBS and SBS–OH, and (**b**) SBS and SBS–Ban_0_, in which “0” means the obtained elastomer without ultraviolet radiation.

The chemical structure of SBS–OH was firstly analyzed by ^1^H NMR. As shown in Figure 1a and Figure S1, in comparison with pristine SBS, new signals were clearly observed in SBS–OH. The peaks at 3.7 ppm and 3.6 ppm were ascribed to the protons (O–CH_2_–CH–O) close to the hydroxyl groups, which indicated that dihydroxyl groups were branched onto the polymer chains and demonstrated the successful preparation of SBS–OH. In addition, based on the integral of the characteristic peaks (3.7–3.6 ppm), the modification ratio relative to PB repeat units could be calculated as 3.06%, which is close to the results (2.93%) obtained from the element analysis. 

When 9-anthraceneboronic acid was mixed with SBS–OH, boronic esters were generated upon heat. As shown in Figure 1b and Figure S2, as a result of the esterification process, the peaks at 8.5 ppm, 8.0 ppm, and 7.5 ppm were ascribed to the appearance of anthracene. It was found that the peak at 3.6 ppm in SBS–OH was divided into two peaks, including 4.5 ppm and 4.7 ppm, while the other characteristic peak was shifted from 3.7 ppm to 5.0 ppm, resulting from the formation of boronic esters. All of these clues suggested that the boronic esters were embedded in the SBS matrix. For convenience, the elastomer of this state without ultraviolet radiation is denoted as SBS–BAn_0_.

**Figure 2:**
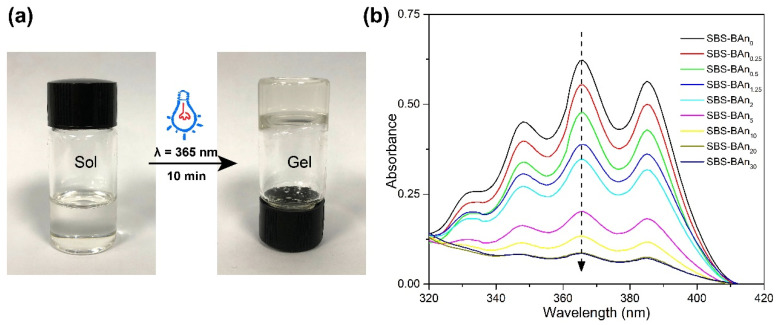
(**a**) Sol–gel transformation of SBS–BAn_0_ after irradiation, and (**b**) UV–vis spectra of SBS–BAn_X_ which was radiated under 365 nm for different times; x is the radiation time.

Then, the reactivity of anthracene in SBS–BAn_x_ was examined preliminarily. In Figure 2a, SBS–BAn_0_ was firstly dissolved in xylene and radiated under a UV lamp (365 nm). Gelation was observed after irradiating for 10 min. According to the sol–gel transformation, the remark can be made that cross-links were formed via the photodimerization of anthracene.

Owing to the absorption of anthracene in the UV–vis spectra, the photodimerization process of anthracene can be monitored in real time. The spectra of SBS–BAn_x_ radiated for different times are shown in Figure 2b; it is obvious that one absorption band around 330–390 nm was observed in the UV–vis spectra, which is the characteristic absorption band of anthracene. Upon being radiated under 365 nm, the absorption intensity of the characteristic absorption band decreased quickly, indicating the photodimerization of anthracene. It is noteworthy that the rate of photodimerization was fast in the first 10 min and there were no significant changes found in the intensity when radiated for 30 min. It is suggested that the photodimerization process of anthracene could be completed in 30 min. Namely, linear SBS–BAn_0_ was changed into cross-linked SBS-BAn_30_ upon 365 nm for 30 min. Therefore, in following experiments, SBS-BAn_30_ was deemed as completely dimerized. According to the SEM image (Figure S3), 9-anthraceneboronic acid is uniformly distributed in the polymer matrix without aggregation, which is attributed to the covalent interactions between 9-anthraceneboronic acid and SBS–OH.

**Figure 3:**
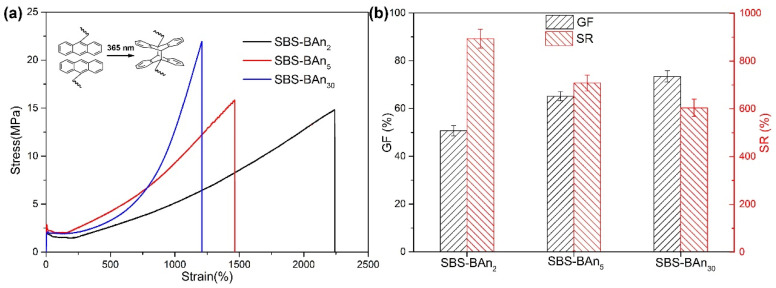
(**a**) Stress–strain curves and (**b**) the gel fraction (GF) and swelling ratio (SR) obtained from swelling tests for SBS–BAn_x_.

Indeed, the cross-linking density increased gradually as a result of the photodimerization of anthracene and could be tailored by irradiating SBS–BAn_x_ for different times. This microscopic variation could also be reflected on the mechanical properties. The stress–strain curves of SBS–BAn_x_ were plotted in Figure 3a; with the radiation time prolonged, the stress increased from 14.83 MPa for SBR-BAn_2_ to 21.94 MPa for SBR-BAn_30_. On the contrary, the strain decreased from 2239% to 1209%. The photodimerization of anthracene increased the cross-links in SBS-BAn_x_ and made the elastomers harder. Owing to the advantages of light, the photodimerization process is gradual and controllable, which is beneficial for the regulation of mechanical properties of the prepared elastomers.

As mentioned above, the solvent resistance of commercial SBS is poor for their physical cross-links. While SBS–BAn_x_ is equipped with chemical cross-links, the solvent resistance has made great progress. Swelling experiments of SBS–BAn_x_ were conducted and the results are summarized in Figure 3b. The results confirmed the existence of networks as well as the solvent resistance. As shown in Figure 3b, the gel fraction (GF) increased from 50% for SBR–BAn_2_ to 73% for SBR-BAn_30_; at the same time, the swelling ratio (SR) decreased from 890% to 600%. It can be concluded that SBS–BAn_x_ chains were cross-linked successfully by the photodimerization of anthracene and the prolongation of radiation time would lead more SBS chains into the networks, which would result in a more insoluble substance.

The mechanical properties of SBS–BAn_x_ in response to UV light based on the photodimerization of anthracene have been discussed. Now, the de-dimerization of anthracene via heat will be studied. Heat inducing the de-dimerization of anthracene would result in a decrease in the cross-linking density in the network, and the mechanical properties would be influenced directly, as revealed by DMA (Figure S4).

**Figure 4:**
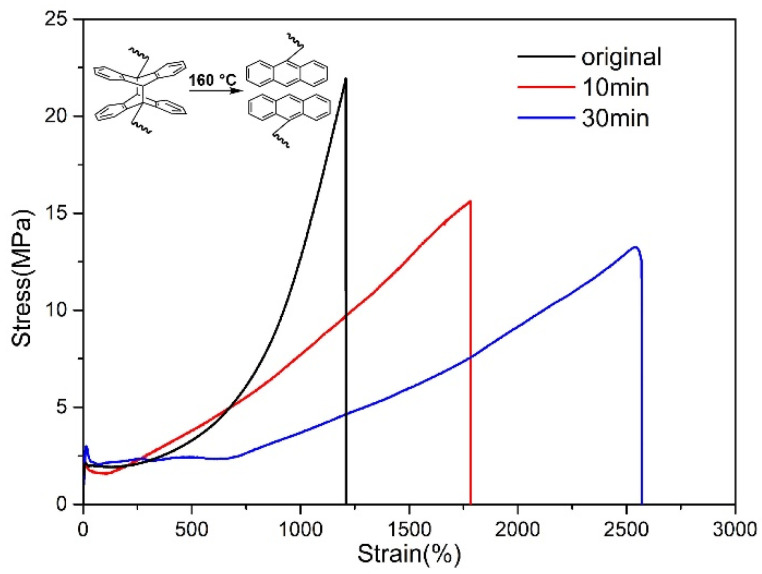
The stress–strain curves of SBS–BAn_30_ heated at 160 °C for different times.

Upon being heated at 160 °C for different times, the mechanical properties were tested and stress–strain curves were plotted in Figure 4. When SBS–BAn_30_ was heated for 10 min, the stress decreased from 21.94 MPa to 15.62 MPa, while the strain increased from 1209% to 1781%. As the heat time prolonged to 30 min, the strain came up to 2547% and kept even, though the sample was treated for a longer time. Notably, when SBS–BAn_30_ had been heated for 30 min and was immersed in xylene, it became soluble compared with the original SBS–BAn_30_. The ^1^H NMR spectrum (Figure S5) of the soluble polymer was similar to SBS–BAn_0_, indicating that heat-induced de-dimerization of anthracene was performed and all the polymer chains were liberated from the network.

**Figure 5:**
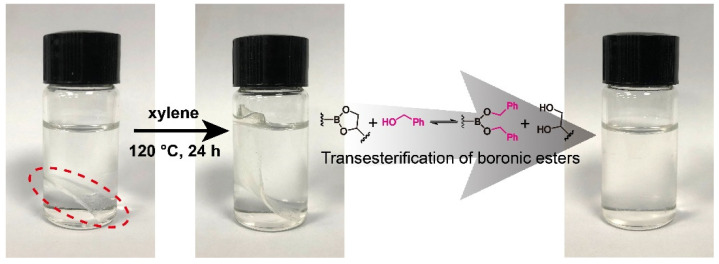
Demonstration of the alcohol response of SBS–BAn_30_ based on boronic esters.

As reviewed [39,40,41], boronic esters are responsive to alcohols. As shown in Figure 5, when pieces were immersed in xylene at 120 °C for 24 h, only the swollen state was observed as expected, indicating that the networks still existed. However, the swollen state dissolved gradually when benzyl alcohol was added into the mixture within 20 min. The dissolved polymer was extracted and analyzed. Its ^1^H NMR spectrum (Figure S6) indicated that it was SBS–OH. Therefore, it can be concluded that benzyl alcohol competed with the hydroxyl groups on SBS–OH to participate in the transesterification of boronic esters. As a result, SBS–OH was liberated from 9-anthraceneboronic acid and the cross-linked network collapsed. Although the transesterification process is entropically disfavored, the equilibrium can be shifted when an excess of benzyl alcohol is used; the molar ratio of benzyl alcohol to boronic esters was kept at 60 in this experiment. The alcohol responses were quite useful; thus, this network can not only be recycled through the stimuli but also act as degradation agents and chemo-sensors for them.

**Figure 6:**
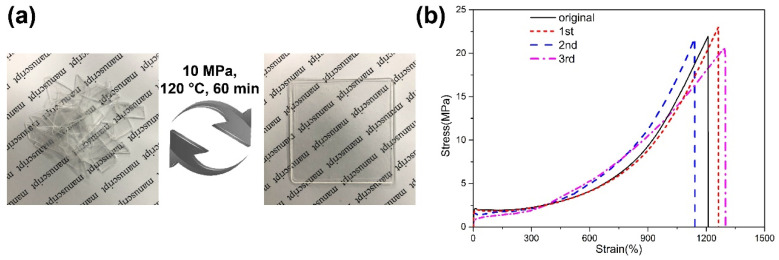
(**a**) Recycling of SBR-BAn_30_ under 10 MPa at 120 °C for 60 min based on the transesterification of boronic esters. (**b**) The comparison of stress–strain for SBR–BAn_30_ after three recycles.

The cross-links would be broken due to the de-dimerization at a high temperature (above 150 °C), providing a reprocessing method for the material. The sample was cut into pieces and molded into a film under 10 MPa at 160 °C for 30 min. As expected, the obtained film was soluble in xylene due to the loss of the network. Nevertheless, the pieces could also be reprocessed at a lower temperature (120 °C), at which the de-dimerization process was demonstrated to be restrained, based on the results of swelling experiments (Figure 6a). This successful reprocessing is attributed to tunable, dynamic boronic esters which were embedded in the network as reported [36,42]. It is worth noting that the network was retained and proved by immersing the resulted film in xylene which was only swollen for days. The DMA results (Figure S4) also demonstrate that the cross-linked networks still existed when the temperature was 120 °C. To quantitatively describe the quality of the recycled samples, the remolding experiment was repeated up to three times and the recycled samples were subjected to tensile tests, as shown in Figure 6b. Compared with the original samples, the acceptable discrepancies also proved the efficiency of the exchange reactions.

It is believed that viscosity is critical for processing [43]. In this contribution, the prepared materials were endowed with two alternative methods by controlling the viscosity in processing. The first one exhibited a sharp viscosity drop for fast processing when the processing temperature was sufficient to activate both the de-dimerization of anthracene and the transesterification of boronic esters. The other one was possessed of Arrhenius-like gradual viscosity variations when the network was only adapted by dynamic boronic esters, such that the processing method can be adjusted according to practical conditions.

## 4. Conclusions

In summary, we reported the preparation of stimuli-responsive elastomers on the basis of two kinds of dynamic components, including boronic esters and anthracene. Commercial SBS was first functionalized with dihydroxyl groups and confirmed via ^1^H NMR, followed by a reaction with 9-anthraceneboronic acid to form boronic esters upon heat. The cross-linked network was constructed as a result of photodimerization of anthracene under ultraviolet radiation (365 nm). The cross-linking density was responsive to radiation time and heating time at 160 °C, which is helpful to regulate the mechanical properties of the prepared elastomers. The recycling of the cross-linked elastomers can be achieved in a manner of network loss on the basis of the de-dimerization of anthracene at 160 °C, or the responsiveness of boronic esters to alcohols. In addition, the recycling could also be implemented under 10 MPa at 120 °C for 60 min. In this way, the network was retained and the mechanical properties of the recycled elastomers were mostly reserved, even after three cycles, as revealed by tensile tests. It is anticipated that the reported synthesis method for SBS elastomers, which exhibited responsiveness to three stimuli and two recycling ways, could be extended to other polymeric matrix and be conducive to exploring more potential applications.

## Data Availability

The data presented in this study are available on request from the corresponding author.

## Supplementary Materials

The following supporting information can be downloaded at: https://www.mdpi.com/article/10.3390/polym14061104/s1, Figure S1: ^1^H NMR spectra of SBS and SBS–OH; Figure S2: ^1^H NMR spectra of SBS and SBS–BAn_0_, in which “0” means the obtained elastomer without ultraviolet radiation; Figure S3:The SEM image of SBS–BAn_30_; Figure S4: The DMA curves of SBS and SBS–BAn_30_; Figure S5: ^1^H NMR spectrum of the resulted polymer after heating SBS-BAn_30_ for 30 min at 160 °C; Figure S6: ^1^H NMR spectrum of the polymer extracted from the benzyl alcohol resulted solution; Table S1: Summarized properties of SBS–BAn_x_; Table S2: Summarized properties of SBS–BAn_30_ heat for different times at 160 °C.
