# Supplementary material for: Integrating Boronic Esters and Anthracene into Covalent Adaptable Networks toward Stimuli-Responsive Elastomers
Source: Polymers (Basel). 2022 Mar 10;14(6):1104. doi: 10.3390/polym14061104 (PMC8954286; doi:10.3390/polym14061104)
Supplement: Supplementary file 1 [file polymers-14-01104-s001.zip › polymers-1556639-supplementary.pdf]

polymers

MDPI

Supporting Information

Integrating Boronic Esters and Anthracene into Covalent Adaptable Networks toward Stimuli-Responsive Elastomers

Zhiyong Liu [1], Youwei Ma [2], Yixin Xiang [1], Xianrong Shen [1], Zixing Shi [2,*] and Jiangang Gao [1,*]

[1] Department of Polymer Materials and Engineering, School of Chemical and Environmental Engineering, Anhui Polytechnic University, Wuhu 241000, China; liuzhiyong@ahpu.edu.cn (Z.L.); xiangyixin@ahpu.edu.cn (Y.X.); shenxianrong@ahpu.edu.cn (X.S.)

[2] State Key Laboratory for Metal Matrix Composite Materials and Shanghai Key Laboratory of Electrical Insulation & Thermal Ageing, School of Chemistry and Chemical Engineering, Shanghai Jiao Tong University, Shanghai 200240, China; mayouwei1995@sjtu.edu.cn

* Correspondence: zxshi@sjtu.edu.cn (Z.S.); gaojiangang@ahpu.edu.cn (J.G.)

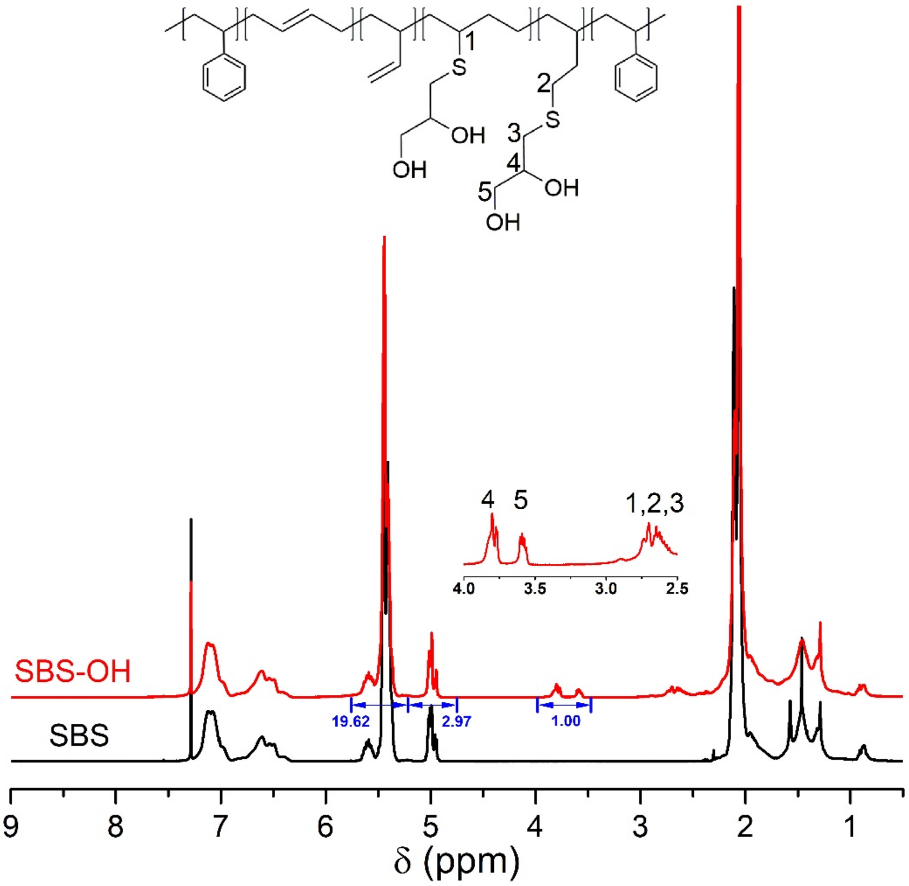

Figure S1. ^{1}H NMR spectra of SBS and SBS-OH.

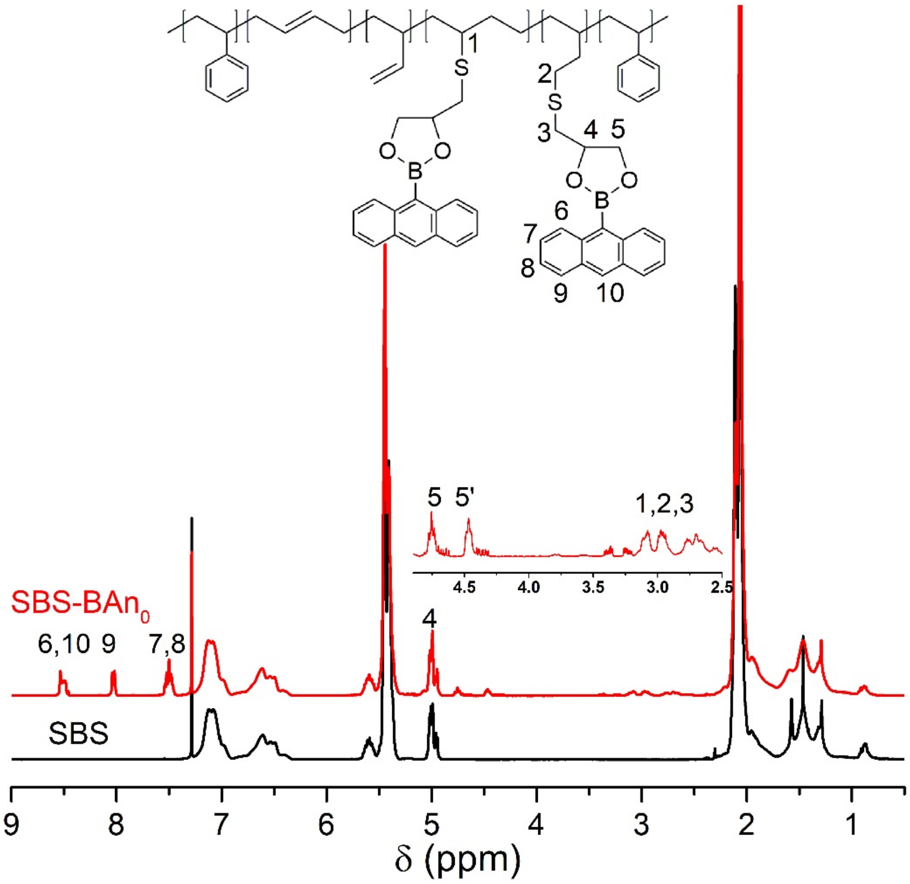

Figure S2. ^{1}H NMR spectra of SBS and SBS-BAn0, in which "0" means the obtained elastomer without ultraviolet radiation.

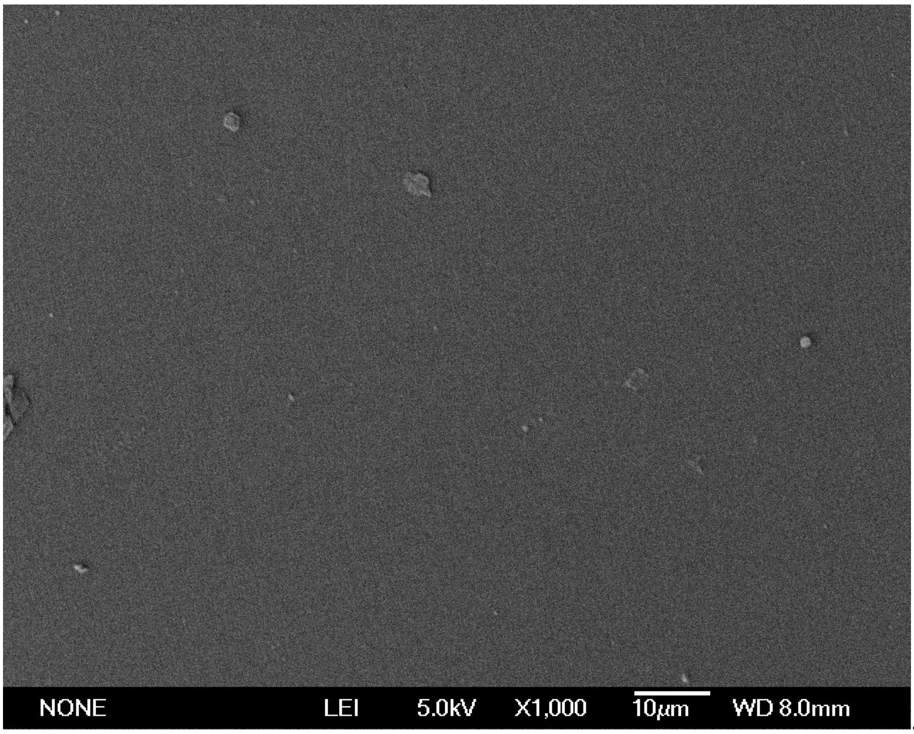

Figure S3. The SEM image of SBS-BAn30.

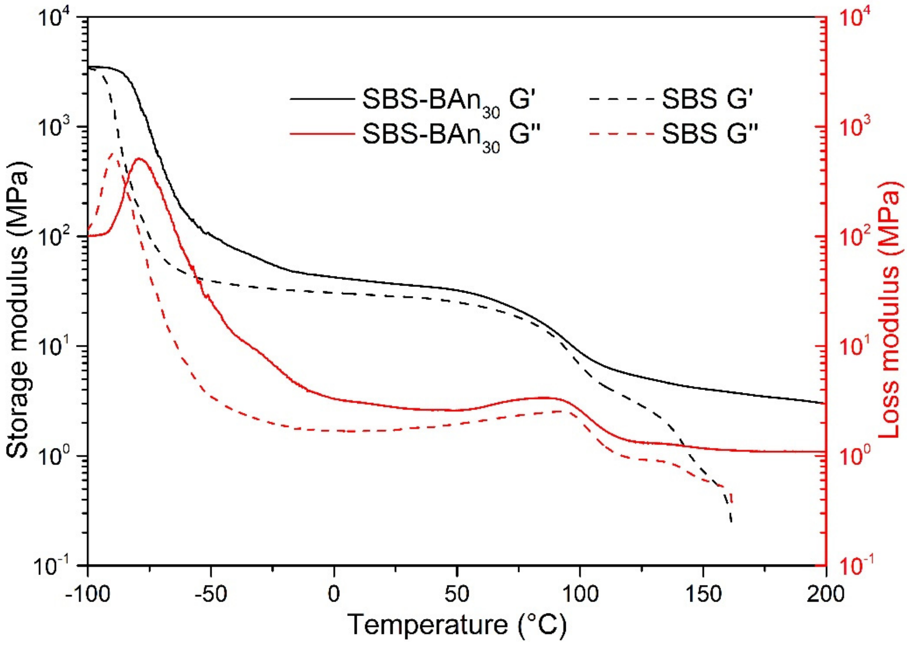

Figure S4. The DMA curves of SBS and SBS-BAn30.

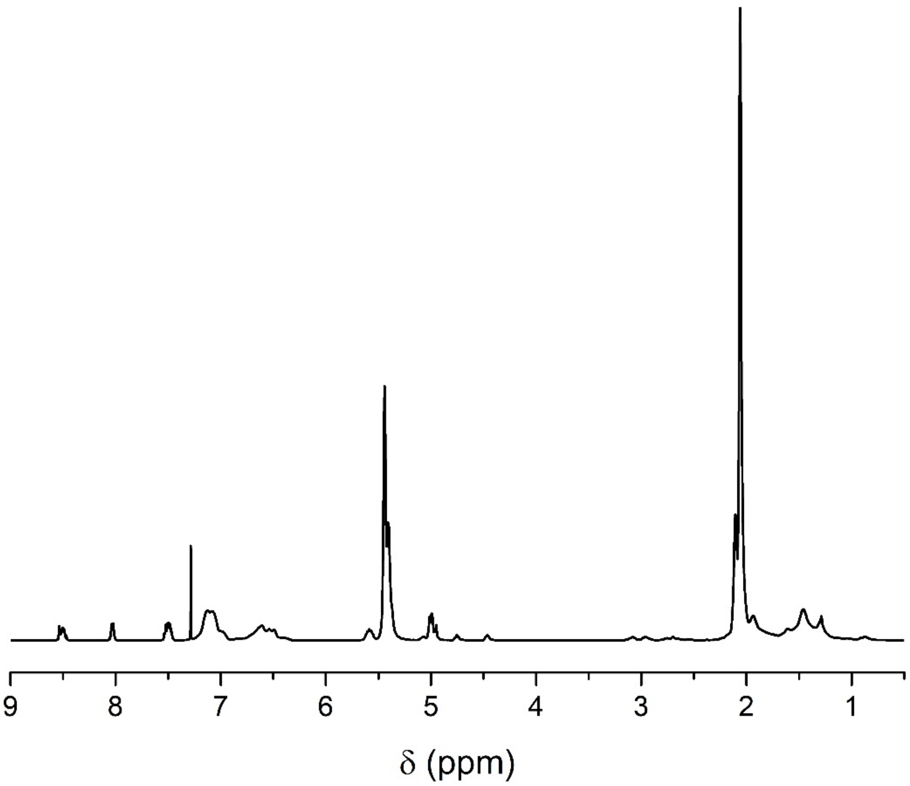

Figure S5. ^{1}H NMR spectrum of the resulted polymer after heating SBS-BAn30 for 30min at 160°C.

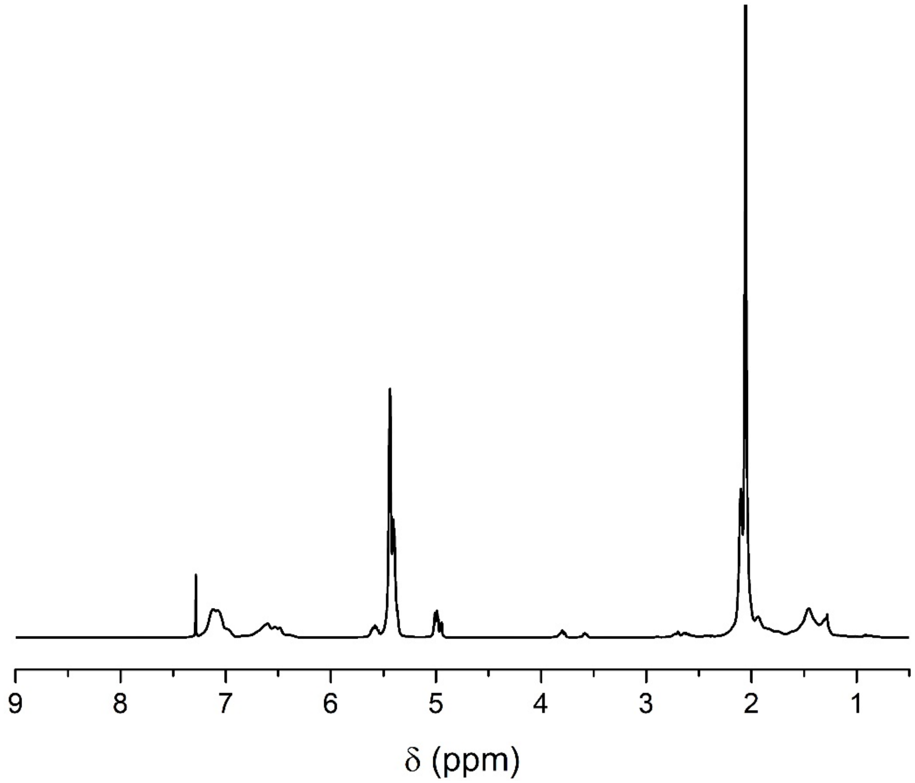

Figure S6. ^{1}H NMR spectrum of the polymer extracted from the benzyl alcohol resulted solution.

Table S1. Summarized properties of SBS-BAn$_x$.

Sample	GF (%)	SR (%)	Strain at break (%)	Stress at break (MPa)
SBS-BAn$_2$	50 ± 2	890 ± 40	2239± 200	14.83 ± 2
SBS-BAn$_5$	65 ± 2	708 ± 33	1463 ± 120	15.83 ± 2
SBS-BAn$_{30}$	73 ± 3	600 ± 36	1209 ± 100	21.94 ± 3

Table S2. Summarized properties of SBS-BAn$_{30}$ heat for different times at 160℃.

Sample	Strain at break (%)	Stress at break (MPa)
Original	1209 ± 100	21.94 ± 3
10min	1781 ± 130	15.62 ± 2
30min	2547 ± 200	13.17 ± 2
